# Development of Prediction Equations for Digestible and Metabolizable Energy in 15 Cereal Processing By-Products Fed to Growing Pigs

**DOI:** 10.3390/ani14213101

**Published:** 2024-10-28

**Authors:** Jinbiao Zhao, Qi Zhu, Xiaoming Song, Meiyu Yang, Ling Liu

**Affiliations:** State Key Laboratory of Animal Nutrition and Feeding, College of Animal Science and Technology, China Agricultural University, Beijing 100193, China; jinbiaozhao@cau.edu.cn (J.Z.);

**Keywords:** cereal processing by-products, growing pigs, available energy, prediction equation, nutrient digestibility

## Abstract

The study was conducted to determine the nutrient digestibility and digestible (DE) and metabolizable energy (ME) of 15 cereal processing by-products and to develop prediction equations for DE and ME based on the chemical composition in growing pigs. A total of 36 crossbred pigs were chosen and divided into three blocks according to a three 12 × 3 Youdin square experimental design. Twelve pigs in each block were allocated into six diets including a basal diet and five test diets. The feeding experiment included three periods, and each dietary treatment contained six replicates. Each period lasted 12 days, including 7 d of dietary adaption and 5 d of total feces and urine collection. The results showed that the coefficients of variation for EE, NDF, ADF, CP, and GE among 15 cereal processing by-products were 86.78%, 49.82%, 51.14%, 14.95%, and 6.26%, respectively. There were significant differences in DE, ME, and ME/DE (DM basis) among 15 cereal processing by-products (*p* < 0.05), ranging from 11.55 MJ/kg to 17.64 MJ/kg, from 10.90 MJ/kg to 17.40 MJ/kg, and from 89.41% to 98.63%, respectively. The digestibility of GE, DM, NDF, ADF, and CP showed a significant difference among 15 cereal processing by-products (*p* < 0.05). The DE and ME contents showed a negative correlation to NDF and ADF (*p* < 0.05), and the prediction equations for DE and ME (as-fed basis) were DE (MJ/kg) = −0.4597 × ADF (%) + 0.5988 × GE (MJ/kg) + 6.0138 (R^2^ = 0.74; *p* < 0.05) and ME (MJ/kg) = 1.0440 × DE (MJ/kg) − 1.1235 (R^2^ = 0.98; *p* < 0.05). On a DM basis, the optimal prediction equations for DE and ME were DE (MJ/kg DM) = −0.1451 × NDF (%) + 0.3026 × CP (%) + 13.8595 (R^2^ = 0.72; *p* < 0.05) and ME (MJ/kg DM) = 1.1155 × DE (MJ/kg DM) + 0.0363 × ADF (%) − 2.3412 (R^2^ = 0.99; *p* < 0.05). In conclusion, there were large variations in the chemical composition, nutrients digestibility, and available energy among the 15 different samples of cereal processing by-products. It is reasonable to develop prediction equations for DE and ME based on the chemical composition of cereal by-products for growing pigs.

## Simple Summary

Many cereal by-products are produced as the development of processing technologies, such as wheat bran, wheat middlings, rice polish, and others. The chemical composition and nutritional value of cereal processing by-products are influenced by the grain varieties and processing techniques. There is limited research on the differences in chemical composition, available energy, and nutrient digestibility among processing by-products derived from corn, wheat, and rice. Additionally, there is a need to develop prediction equations for available energy based on their varying chemical compositions. This study selected 15 cereal processing by-products to assess their nutrient digestibility and available energy contents together and to develop prediction equations for digestible energy and metabolizable energy based on their chemical composition in growing pigs.

## 1. Introduction

In 2023, China’s annual output of corn, wheat, and rice was 289, 137, and 207 million tons, respectively, according to the published data from the National Bureau of Statistics of China (https://data.stats.gov.cn/easyquery.htm?cn=C01 accessed on 10 September 2024). Many by-products are produced in the process of cereal deep processing, such as wheat bran, wheat middlings, rice polish, rice bran, corn bran, corn germ meal, and distillers dried grains with solubles (DDGS). Among them, the annual output of wheat bran, rice bran, and DDGS is 26, 10, and 7 million tons, respectively [1]. Cereal processing by-products are always used as energy-supplied feed ingredients because they contain a lot of digestible nutrients, such as protein, starch, ether extract, and fermentable fiber components [2]. In a word, there are abundant resources of cereal processing by-products, but the chemical composition and nutritional value of cereal processing by-products from different sources vary due to the differences in grain planting varieties and processing techniques [3,4].

Variations in the chemical composition and nutritional values among different samples of the single cereal processing by-product have been widely studied. The publications had built the prediction equations for digestible energy (DE), metabolizable energy (ME), and standardized ileal amino acid digestibility in wheat processing by-products for growing pigs [5,6]. Huang et al. [7] compared the available energy and apparent and standardized ileal digestibility of crude protein and amino acids in nine samples of defatted rice bran fed to growing pigs and generated prediction equations for DE and ME based on the chemical composition. Some previous studies reported the determination of the energy contents of corn distillers dried grains with solubles containing different levels of oil in growing pigs and developed the prediction equations for DE and ME basal on the chemical composition [8,9,10]. Wang et al. [11] developed the prediction equations for DE and ME based on the chemical composition of Chinese corn gluten feed fed to growing-finishing pigs. However, there are few publications studying the differences in the chemical composition, available energy, and nutrient digestibility among different cereal processing by-products derived from corn, wheat, and rice and exploring the prediction equations for available energy based on their varying chemical compositions. In addition, different processing methods would affect the energy contents, chemical composition, and nutrient digestibility. As feed processing technologies develop, it is of significance to determine the energy contents and chemical composition of cereal processing by-products, which are produced in the present processing methods.

The hypothesis of this study was that the prediction equations for available energy based on the chemical composition of different kinds of cereal processing by-products can be developed quickly and effectively in the pig production. Therefore, a total of 15 cereal processing by-products were chosen to determine the nutrient digestibility and DE and ME contents in this study and to develop prediction equations for DE and ME based on their chemical composition in growing pigs

## 2. Materials and Methods

The digestion and metabolism trial of pigs was conducted at FengNing Swine Research Unit of China Agricultural University (Academician Workstation in Chengdejiuyun Agricultural and Livestock Co., Ltd., Fengning, China). The study was approved by the Institutional Animal Care and Use Committee of China Agricultural University (CAU AW31604202–1-2, Beijing, China).

### 2.1. Animals, Diets, and Experimental Design

A total of 36 healthy Duroc × Landrace × Yorkshire crossbred pigs with an initial body weight of 38.23 ± 1.42 kg were chosen and divided into three blocks randomly according to a three 12 × 3 Youdin square experimental design. Twelve pigs in each block were allocated into six diets including a basal diet and five test diets, and the test diets were formulated with one of the cereal by-products replacing 30% of the energy supply components of the basal diet (Table 1). Each dietary treatment contained six replicates and one pig per replicate. The feeding experiment included three periods and each period lasted 12 d, including 7 d of dietary adaption and 5 d of total feces and urine collection. The vitamins and trace minerals were supplemented in the diets to meet the swine feeding standards [12], and the composition and nutrient levels of the diets are shown in the Table 2.

### 2.2. Feeding Management

The pigs were placed in individual stainless steel metabolism cages (1.3 m × 0.6 m × 0.8 m) on concrete slatted floors and within temperature-controlled rooms. The cages were equipped with a feeder at the front of the cage and a nipple drinker on one side to limit feed spillage and keep the feed separate from the water. The temperature and humidity of the pig house were controlled at 22 ± 2 °C and 60 ± 3%, respectively. Feed was offered to the pigs at 08:30 and 15:30 h, in a daily amount of 4% of pig body weight in the experiment. The pigs had free access to water throughout the feeding trial.

### 2.3. Samples Collection and Analysis

During fecal and urine collection, the leftovers and sprinkles were collected before and after feeding and then weighed and recorded after drying. Two stainless steel trays were placed under each pig cage. A plastic bucket was placed under the front stainless-steel tray for urine collection, and the rear stainless-steel tray was placed for feces collection. Before collecting the urine sample, 10 mL of 6 mol/L hydrochloric acid was added to 1 L of urine to fix nitrogen. The volume of daily urine was recorded every day, and 10% of the urine was collected in a plastic bottle and stored at −20 °C. The urine samples of each pig for 5 days were mixed, and 10% were filtered with gauze and stored at −20 °C in a 50 mL centrifuge tube for further analysis. All feces from each pig were collected every six hours and stored at −20 °C after collection. After the end of each period, all the feces collected from one pig was thawed, weighed, and mixed evenly. Then, two trays of 350 g of feces were taken and then dried in an oven at 65 °C for 72 h and moistened for 24 h. After crushing, the fecal samples were screened via 40 mesh and bagged for further analysis.

All samples of ingredients, diets, and feces were ground through a 1 mm screen and analyzed for dry matter (DM) (Method 927.05, AOAC), CP (Method 990.03, AOAC), ash (Method 975.03, AOAC), neutral detergent fiber (NDF) and acid detergent fiber (ADF) (Method 973.18, AOAC), and ether extract (Method 920.39) [13]. The gross energy (GE) of the ingredients, diets, feces, and urine was determined by an Adiabatic Oxygen Bomb Calorimeter (Parr Instruments, Moline, IL, USA), with benzoic acid (6318 kcal GE/kg; Parr Instrument Co., Moline, IL, USA) as the standard [14]. Then, 4 mL of urine was added to filter paper in the bomb crucible and dried at 55 °C for 24 h before determining GE. An analysis of urine energy was conducted in triplicate.

### 2.4. Calculation

(1) Nutrient digestibility in the diets was calculated as follows:

AD_diet_ (%) = (DN_intake_ − FN_excretion_)/DN_intake_ × 100

where AD _diet_ (%) represents nutrient digestibility in the diet; DN _intake_ represents the intake of dietary nutrients; FN _excretion_ represents the excretion of fecal nutrients.

(2) The DE and ME in the diets were calculated as following:

Dietary DE (MJ/kg) = (GE_intake_, MJ − FE_excretion_, MJ)/Feed intake, kg

Dietary ME (MJ/kg) = (GE_intake_, MJ − FE_excretion_, MJ − UE_excretion_, MJ)/Feed intake, kg

where GE _intake_ represents the intake of total dietary gross energy; FE _excretion_ represents the excretion of total gross energy in the feces; and UE _excretion_ represents the excretion of total gross energy in the urine.

(3) Nutrient digestibility in the test cereal processing by-products was calculated as follows [15]:

AD_ingredient_ (%) = (FN_test diet_ × FD_test diet_ − FN_basal diet_ × FD_basal diet_ × (1 − X%))/(AN_ingredient_ × X%)

where AD_ingredient_ represents nutrient digestibility in the test feed ingredients; FN_test diet_ represents the nutrient level in the test diets; FD _test diet_ represents nutrient digestibility in the test diets; FN_basal diet_ represents the nutrient level in the basal diets; FD_basal diet_ represents nutrient digestibility in the basal diets; AN_ingredient_ represents the nutrient level in the test feed ingredients; and X% represents a proportion of test feed ingredients accounted for in the energy supplier in the basal diet.

(4) The DE and ME in the test cereal processing by-products were calculated as follows [16]:

DE_ingredient_ (MJ/kg) = (DE_test diet_ (MJ/kg) − DE_basal diet_ (MJ/kg) × (1 − X%))/X%

ME_ingredient_ (MJ/kg) = (ME_test diet_ (MJ/kg) − ME_basal diet_ (MJ/kg) × (1 − X%))/X%

where DE_ingredient_ and ME_ingredient_ represent DE and ME in the test feed ingredients; DE_test diet_ and ME_test diet_ represent DE and ME in the test diets; DE_basal diet_ and ME_basal diet_ represent DE and ME in the basal diets; and X% represents a proportion of test feed ingredients accounted for in the energy supplier in the basal diet.

### 2.5. Statistical Analyses

The data were checked for normality, outliers were detected using the UNIVARIATE procedure of SAS (SAS Institute 9.4, Cary, NC, USA), and no outliers were identified in the present study. The individual pig was considered as the experimental unit. The data were analyzed by the GLIMMIX procedure using the Mixed Procedure as a randomized complete block design. The fixed effect was the dietary treatment, and the block and period were considered as random effects. Treatment means were calculated using the LSMEANS statement, and statistical differences among the treatments were separated by Tukey’s test. The correlation coefficients of the available energy and chemical composition of the test cereal processing by-products were analyzed using the PROC CORR program. The prediction equations for the DE and ME of test feed ingredients were established by the Stepwise Regression method of the PROC REG program. The coefficient of determination (R^2^), Mallows’Cp (Cp), Akakike information criterion (AIC), and Bayesian information criterion (BIC) were used to evaluate the optimal prediction equation [17] and the maximum R^2^ and minimum C (p). AIC and BIC represent the best prediction model. Statistical significance was declared at *p* < 0.05, and a tendency was considered when 0.05 ≤ *p* < 0.10.

## 3. Results

### 3.1. Chemical Composition in 15 Cereal Processing By-Products

The concentrations of DM, ash, EE, NDF, ADF, CP, and GE in the 15 samples of cereal processing by-products are shown in Table 3. The results show that the averages of DM, ash, EE, NDF, ADF, CP, and GE were 89.63%, 4.90%, 6.49%, 25.92%, 7.50%, 16.81%, and 17.49 MJ/kg (as-fed basis) with CV values of 1.59%, 49.61%, 86.78%, 49.82%, 51.14%, 14.95%, and 6.24% respectively.

### 3.2. Available Energy and Nutrients Digestibility in the Diets

The DE, ME, and nutrient digestibility of all test diets were shown in Table 4. The results showed that there were significant differences in the DE, ME, and digestibility of GE, DM, ash, NDF, ADF, and CP among 15 different test diets (*p* < 0.05). The contents of DE, ME, and ME/DE in the diets (as-fed basis) ranged from 12.59 to 14.34 MJ/kg, from 12.28 to 14.07 MJ/kg, and from 95.75% to 98.25%, with averages of 13.42 MJ/kg, 13.05 MJ/kg, and 97.22%, respectively.

### 3.3. Available Energy and Nutrients Digestibility in the Cereal Processing By-Products

The DE and ME contents in 15 cereal processing by-products are shown in Table 5. The results show that there were significant differences in the DE, ME, and ME/DE among the 15 cereal processing by-products (*p* < 0.05). The contents of DE and ME (on a DM basis) and the ratio of ME to DE ranged from 11.55 MJ/kg to 17.64 MJ/kg, from 10.90 MJ/kg to 17.40 MJ/kg, and from 89.41% to 98.63%, with averages of 14.56 MJ/kg, 13.95 MJ/kg, and 95.62%, respectively.

The nutrient digestibility of 15 cereal processing by-products is shown in Table 6. The results show that there were significant differences in the digestibility of GE, DM, NDF, ADF, and CP among the 15 cereal processing by-products (*p* < 0.05). The digestibility of GE, DM, NDF, ADF, and CP ranged from 60.73% to 87.71%, from 56.33% to 90.68%, from 42.03% to 80.31%, from 27.46% to 57.41%, and from 44.69% to 96.09%, with averages of 74.93%, 73.03%, 60.15%, 41.72%, and 77.37%, respectively.

### 3.4. Prediction Equations for DE and ME

The correlations between the available energy contents and chemical composition for the 15 cereal processing by-products in growing pigs are shown in Table 7. The results showed that the DE and ME had significant negative correlations with the concentrations of NDF and ADF (*p* < 0.05). And the ME showed a significant positive correlation with the DE content (*p* < 0.05). The prediction equations for the DE and ME (as-fed basis) of the test cereal processing by-products are shown in Table 8. The prediction equations for DE were developed as follows: DE (MJ/kg) = −0.4110 × ADF (%) + 16.1184 (R^2^ = 0.74; *p* < 0.05) and DE (MJ/kg) = −0.4597 × ADF (%) + 0.5988 × GE (MJ/kg) + 6.0138 (R^2^ = 0.86; *p* < 0.05). And a prediction equation for ME (as-fed basis) was also built: ME (MJ/kg) = 1.0440 × DE (MJ/kg) − 1.1235 (R^2^ = 0.98; *p* < 0.05). On a DM basis, the optimal prediction equations for DE and ME were DE (MJ/kg DM) = −0.1451 × NDF (%) + 0.3025 × CP (%) +13.8595 (R^2^ = 0.72; *p* < 0.05) and ME (MJ/kg DM) = 1.1155 × DE (MJ/kg DM) + 0.0363 × ADF (%) − 2.34115 (R^2^ = 0.99; *p* < 0.05).

## 4. Discussion

### 4.1. Chemical Composition

The data for the chemical compositions of the different cereal processing by-products in the present study were consistent with the previous reports [18,19,20]. The nutritional values including the available energy contents and nutrient digestibility are closely associated with the chemical composition of cereal processing products.

### 4.2. Available Energy and Nutrients Digestibility

A previous study reported the effects of different inclusion levels (15%, 25%, 35%, 45%, and 55%) and adaptation periods (7 d, 14 d, 21 d, and 28 d) on the DE content and the apparent total tract digestibility (ATTD) of GE and CP for wheat bran, and the results showed that the DE and digestibility of GE and CP ranged from 10.35 MJ/kg to 11.39 MJ/kg (as-fed basis), from 63.3% to 71.1%, and from 67.0% to 86.3%, respectively [21]. The results mentioned above were consistent with our findings, in which they were from 10.30 MJ/kg to 11.50 MJ/kg, from 60.73% to 67.08%, and from 72.20% to 80.48%, respectively.

A previous study determined the DE and ME contents of 19 rice bran samples and developed prediction equations for DE and ME based on their chemical composition, and the results showed that the DE, ME, and ME/DE (DM basis) were from 14.48 MJ/kg to 16.85 MJ/kg, from 12.49 MJ/kg to 15.84 MJ/kg, and from 84.49% to 96.00%, respectively [22]. However, our finding showed that the DE and ME contents among five different samples of rice bran ranged from 12.66 MJ/kg to 16.63 MJ/kg and from 11.32 MJ/kg to 16.08 MJ/kg. The differences in DE and ME among different rice bran samples are associated with the content of fat, because there is a large variation in the fat content in rice bran due to the different processing technologies [7].

Shi et al. [23] determined the DE and ME contents of 10 different corn germ meal samples and developed equations to predict the corresponding energy contents based on the chemical characteristics of corn germ meal, and the results showed that the content of DE and ME (DM basis) and the digestibility of GE in corn germ meals ranged from 10.22 MJ/kg to 15.83 MJ/kg, from 9.94 MJ/kg to 15.43 MJ/kg, and from 54.12% to 72.99%, respectively. The above publication was consistent with our findings that the DE, ME, and GE digestibility ranged from 11.76 MJ/kg to 12.89 MJ/kg, from 10.90 MJ/kg to 12.20 MJ/kg, and from 60.77% to 67.16%, respectively. He et al. [24] reported that the DE and ME in fermented corn germ meal were 13.97 MJ/kg and 13.26 MJ/kg, which are higher compared with the results for corn germ meal in our study. The reason for the above observation should be associated with the lower fiber component contents (with a reduction of 30%) and improved nutrient digestibility after the in vitro microbial fermentation.

There were large differences in the contents of crude fiber, protein, and starch among different samples of rich polish due to the different processing technologies, resulting in variations in the DE, ME, and nutrients digestibility. A previous study reported the chemical compositions and ME contents of 20 different types of rice polish, and the results showed that the ME content among 20 samples of rice polish ranged from 5.53 MJ/kg to 12.90 MJ/kg [25]. However, in the present study, the ME contents in two different sources of rice polish were 15.04 MJ/kg and 15.35 MJ/kg, which were greater than those in the previous report mentioned above [25].

Overall, the results for the available energy contents and nutrients digestibility in the different samples of cereal processing by-products were consistent with the previous reports mentioned above [25]. The different available energy contents and nutrient digestibility among different cereal processing by-products were also associated with the inclusion levels in the diet, the dietary adaption period for pigs, and the body weight of pigs, as well as the varying chemical compositions due to the different cereal origin and processing technologies [26,27,28]. Generally, increasing the inclusion levels of ingredients can reduce the risk of calculation errors when determining the nutrient and energy digestibility of high-fiber feed ingredients [29,30].

In addition, our findings showed that samples of wheat middlings, rice polish, and rice bran have greater contents of DE and ME and a greater digestibility of GE compared with wheat bran and corn germ meal, which was consistent with the previous study [31]. The reason for the greater available energy and energy digestibility in wheat middlings, rice polish, and rice bran should be the lower content of fiber components, which is always considered as an anti-nutritional factor for decreasing nutrient digestion and absorption [32]. In addition, wheat middlings and rice polish have a high content of starch, which is moreeasily digested and supplies more energy for the pigs [33].

### 4.3. Prediction Equations for DE and ME

The current results showed that the DE and ME had significant negative correlations with the concentrations of NDF and ADF, and the ME showed a significant positive correlation with the DE content. The observations for a negative correlation between the available energy and fiber components were consistent with the previous studies using samples of wheat, barley, or corn co-products [34,35,36,37]. The observations for a positive correlation between DE and ME were consistent with the previous studies using a single sample of corn, soybean meal, or rapeseed meal, respectively [38,39,40]. Prediction equations for single cereal processing byproducts based on the chemical composition have also been developed. Shi et al. [23] developed equations for predicting DE and ME contents based on the chemical composition in the samples of 10 corn germ meals, and the optimal prediction equations were DE (MJ/kg DM) = 26.85 − 0.28 × IDF (insoluble dietary fiber, %)–17.79 × Calcium (%) and ME (MJ/kg DM) = 0.40 + 0.93 × DE (MJ/kg DM). Shi et al. [22] reported that the prediction equations for DE and ME of rice bran using 17 different samples were DE (MJ/kg DM) = 7.78 − 0.09 × CP + 0.25 × AEE + 0.05 × Starch − 0.08 × NDF + 0.29 × ADF − 0.13 × Ash − 1.8 × Calcium + 2.43 × Phosphorus and ME (MJ/kg DM) = 5.00 + 0.04 × CP + 0.32 × AEE (acid-hydrolyzed ether extract) + 0.05 × Starch − 0.11 × NDF + 0.32 × ADF + 0.19 × Ash − 5.56 × Calcium + 0.23 × Phosphorus. Huang et al. [40] reported that the prediction equations for the DE and ME of wheat middlings were DE (MJ/kg DM) = −0.13 × NDF (%) + 16.92 and ME (MJ/kg DM) = −0.6 × NDF% − 0.16 × xylan (%) + 0.26 × CP (%) − 2.02 × Phosphorus (%) + 12.73. Yang et al. [41] reported that the best-fit equations for wheat bran were DE (MJ/kg DM) = −7.09 + 1.54 × CP (%) − 0.25 × NDF (%) − 0.32 × ADF (%) + 0.23 × Ash (%) and ME (MJ/kg DM) = 0.02 + 0.96 × DE (MJ/kg DM). Considering the prediction equations for DE and ME using 15 cereal processing by-products in our study, more predictive factors should be considered to be used for developing prediction equations, such as starch, non-starch polysaccharides, and soluble fibers.

## 5. Conclusions

The findings indicated that there were large variations in the chemical composition, nutrients digestibility, and available energy among 15 different samples of cereal by-products. The best prediction equations for the DE and ME (as-fed basis) of cereal by-products in growing pigs were DE (MJ/kg) = −0.4597 × ADF (%) + 0.5988 × GE (MJ/kg) + 6.0138 and ME (MJ/kg) = 1.0440 × DE (MJ/kg) − 1.1235. On a DM basis, the optimal prediction equations for DE and ME were DE (MJ/kg DM) = −0.1451 × NDF (%) + 0.3026 × CP (%) + 13.8595 and ME (MJ/kg DM) = 1.1155 × DE (MJ/kg DM) + 0.0363 × ADF (%) − 2.3412. Generally, the nutrient digestibility and available energy in wheat middlings, rich polish, and rice bran were higher than those in the wheat bran and corn germ meal.

## Figures and Tables

**Table 1:** Composition of dietary formulation (As-fed basis, %).

Items	Basal Diet	Test Diet
Corn	97.40	68.18
Test cereal by-products	-	29.22
Dicalcium phosphate	0.90	0.90
Limestone	0.90	0.90
NaCl	0.30	0.30
Premix ^1^	0.50	0.50
Total	100.00	100.00

Note: ^1^ Premix provided the following per kg of a complete diet for growing pigs: vitamin A, 5512 IU; vitamin D_3_, 2200 IU; vitamin E, 30 IU; vitamin K_3_, 2.2 mg; vitamin B_12_, 27.6 μg; riboflavin, 4.0 mg; pantothenic acid, 14.0 mg; niacin, 30.0 mg; choline chloride, 400.0 mg; folacin, 0.7 mg; thiamine 1.5 mg; pyridoxine 3.0 mg; biotin, 44.0 ug; Mn, 40.0 mg; Fe, 75.0 mg; Zn, 75.0 mg; Cu, 100.0 mg; I, 0.3 mg; Se, 0.3 mg.

**Table 2:** Chemical composition of the diets (As-fed basis, %).

	Diets														
Items	Wheat Middlings			Rice Polish		Rice Bran					Wheat Bran			Corn Germ Meal	
	1	2	3	1	2	1	2	3	4	5	1	2	3	1	2
DM	87.31	87.96	87.29	89.83	88.89	88.30	88.41	87.98	88.14	82.28	89.67	87.20	87.41	90.12	87.75
Ash	3.99	4.17	3.50	5.46	3.45	5.06	3.36	5.26	3.36	4.83	4.30	2.98	3.28	3.24	2.87
EE	2.52	1.86	2.82	1.48	2.54	4.92	3.53	7.27	2.01	6.69	1.52	2.47	2.54	1.72	1.77
NDF	13.77	13.46	11.01	11.63	6.87	10.86	9.94	13.10	7.29	12.32	15.54	16.80	19.46	19.97	20.46
ADF	3.31	2.41	2.84	4.01	1.50	3.60	3.47	4.16	1.53	4.48	4.29	4.45	5.55	5.08	5.42
CP	10.83	11.04	11.25	10.75	9.56	9.33	9.36	12.48	8.82	9.46	10.89	10.99	10.86	10.74	12.24
GE, MJ/kg	15.89	15.85	15.82	15.71	15.96	16.38	16.32	16.73	15.82	15.53	15.97	15.88	16.02	16.17	15.96

Analysis conducted in duplicate. DM, dry matter; EE, ether extract; NDF, neutral detergent fiber; ADF acid detergent fiber; CP, crude protein; GE, gross energy.

**Table 3:** Chemical composition of test cereal processing by-products (%, as-fed basis).

Items	Wheat Middlings			Rice Polish		Rice Bran					Wheat Bran			Corn Germ Meal		CV, %
	1	2	3	1	2	1	2	3	4	5	1	2	3	1	2	
DM	88.30	87.94	87.76	88.50	88.24	90.42	90.24	90.92	88.94	90.90	91.16	89.22	89.15	92.69	90.00	1.59
Ash	3.26	3.96	3.13	3.08	2.85	7.63	5.27	10.13	9.50	4.11	4.88	4.94	5.47	1.91	3.34	49.61
EE	3.84	4.56	4.54	5.09	3.03	12.42	11.03	18.07	2.02	18.07	1.72	4.43	3.80	3.39	1.28	86.78
NDF	22.89	20.04	22.60	7.37	3.71	20.00	20.96	23.57	21.21	20.71	32.99	35.55	45.62	48.01	43.50	49.82
ADF	5.54	4.42	4.26	0.86	1.01	7.52	5.36	9.59	8.36	8.82	9.70	9.60	12.98	12.04	12.49	51.14
CP	17.28	17.69	18.51	14.47	12.08	15.57	13.04	19.87	16.13	14.44	17.43	18.19	17.61	18.58	21.22	14.95
GE, MJ/kg	16.72	16.76	16.81	17.09	16.41	18.44	18.29	19.73	15.94	19.51	17.13	17.29	16.95	17.93	17.29	6.24

Analysis conducted in duplicate. DM, dry matter; EE, ether extract; NDF, neutral detergent fiber; ADF acid detergent fiber; CP, crude protein; GE, gross energy.

**Table 4:** Available energy and nutrient digestibility of test diets for growing pigs (As-fed basis).

Items	Wheat Middlings Diets			Rice Polish Diets		Rice Bran Diets					Wheat Bran Diets			Corn Germ Meal Diets		SEM	*p*-Value
	1	2	3	1	2	1	2	3	4	5	1	2	3	1	2		
Available energy, MJ/kg																	
DE	13.56 ^ab^	13.83 ^ab^	13.85 ^ab^	14.30 ^a^	14.34 ^a^	13.70 ^ab^	14.05 ^ab^	13.90 ^ab^	13.08 ^ab^	12.59 ^b^	12.97 ^ab^	12.90 ^ab^	12.63 ^b^	12.80 ^b^	12.85 ^b^	0.37	0.01
ME	13.14 ^abc^	13.42 ^abc^	13.53 ^abc^	13.98 ^a^	14.07 ^a^	13.40 ^abc^	13.72 ^ab^	13.49 ^ab^	12.53 ^bc^	12.37 ^bc^	12.63 ^bc^	12.50 ^bc^	12.28 ^c^	12.29 ^c^	12.44 ^bc^	0.32	0.01
ME/DE	96.88 ^ab^	97.02 ^ab^	97.65 ^ab^	97.75 ^ab^	98.11 ^a^	97.79 ^ab^	97.65 ^ab^	97.04 ^ab^	95.75 ^b^	98.25 ^a^	97.37 ^ab^	96.91 ^ab^	97.23 ^ab^	96.06 ^ab^	96.82 ^ab^	0.58	0.01
Nutrients digestibility, %																	
GE	85.35 ^c^	87.25 ^bc^	87.55 ^b^	89.61 ^a^	90.33 ^a^	83.63 ^cd^	86.13 ^bc^	83.06 ^def^	83.29 ^d^	81.04 ^ef^	81.20 ^ef^	81.25 ^ef^	78.84 ^f^	79.14 ^f^	80.51 ^f^	0.46	0.01
DM	86.09 ^bc^	87.98 ^ab^	87.93 ^ab^	89.88 ^a^	90.72 ^a^	82.73 ^d^	85.48 ^c^	82.60 ^d^	82.53 ^d^	81.39 ^de^	82.42 ^d^	81.98 ^d^	79.33 ^e^	80.96 ^de^	81.69 ^d^	0.53	0.01
Ash	46.12 ^b^	50.98 ^a^	43.93 ^b^	42.05 ^bc^	44.06 ^b^	29.22 ^d^	30.27 ^d^	38.39 ^c^	32.57 ^d^	52.55 ^a^	45.78 ^b^	13.59 ^f^	24.87 ^e^	38.99 ^c^	22.57 ^f^	1.24	0.01
NDF	58.72 ^ab^	61.87 ^a^	61.51 ^a^	57.62 ^ab^	61.35 ^a^	39.20 ^e^	47.45 ^cd^	56.81 ^ab^	45.60 ^d^	53.04 ^bc^	48.87 ^cd^	56.92 ^ab^	48.25 ^cd^	54.19 ^bc^	59.63 ^ab^	1.02	0.01
ADF	48.87 ^bc^	40.44 ^de^	54.41 ^a^	42.59 ^cd^	43.11 ^cd^	33.22 ^e^	40.07 ^de^	46.73 ^bc^	37.39 ^e^	48.90 ^bc^	35.90 ^e^	39.36 ^de^	38.20 ^e^	50.70 ^bc^	56.16 ^a^	0.89	0.01
CP	80.25 ^bc^	84.19 ^a^	83.43 ^ab^	80.90 ^abc^	82.03 ^abc^	72.68 ^fg^	77.11 ^cde^	77.86 ^cde^	74.57 ^ef^	68.01 ^g^	76.55 ^cde^	79.08 ^cd^	74.80 ^ef^	69.71 ^g^	71.71 ^fg^	0.75	0.01

Means within a row with different letters differ among different dietary treatments (*p* < 0.05), *n* = 6. DE, digestible energy; ME, metabolizable energy; DM, dry matter; EE, ether extract; NDF, neutral detergent fiber; ADF acid detergent fiber; CP, crude protein; GE, gross energy.

**Table 5:** Digestible and metabolizable energy of cereal processing by-products for growing pigs.

Items	Wheat Middlings			Rice Polish		Rice Bran					Wheat Bran			Corn Germ Meal		SEM	*p*-Value
	1	2	3	1	2	1	2	3	4	5	1	2	3	1	2		
As-fed basis, MJ/kg																	
DE	13.49 ^abc^	14.40 ^ab^	14.44 ^ab^	15.43 ^a^	15.56 ^a^	13.79 ^abc^	15.01 ^a^	14.61 ^ab^	11.26 ^cd^	12.01 ^bcd^	11.50 ^cd^	11.22 ^cd^	10.30 ^d^	10.90 ^cd^	11.60 ^cd^	0.78	0.01
ME	12.80 ^abc^	13.74 ^ab^	14.02 ^ab^	15.04 ^a^	15.35 ^a^	13.39 ^abc^	14.51 ^a^	13.89 ^ab^	10.07 ^cd^	11.66 ^bcd^	11.26 ^bcd^	10.60 ^cd^	9.86 ^d^	10.10 ^cd^	10.98 ^bcd^	0.72	0.01
ME/DE	94.85 ^ab^	95.43 ^ab^	97.08 ^a^	97.47 ^a^	98.63 ^a^	97.06 ^a^	96.69 ^a^	95.10 ^ab^	89.41 ^b^	97.02 ^a^	97.92 ^a^	94.54 ^ab^	95.70 ^a^	92.70 ^ab^	94.66 ^ab^	1.83	0.01
DM basis, MJ/kg																	
DE	15.28 ^abc^	16.37 ^ab^	16.46 ^ab^	17.43 ^a^	17.64 ^a^	15.25 ^abc^	16.63 ^ab^	16.07 ^ab^	12.66 ^bc^	13.22 ^bc^	12.61 ^bc^	12.57 ^bc^	11.55 ^c^	11.76 ^c^	12.89 ^bc^	0.86	0.01
ME	14.49 ^abcd^	15.62 ^ab^	15.98 ^ab^	16.99 ^a^	17.40 ^a^	14.81 ^abc^	16.08 ^ab^	15.28 ^ab^	11.32 ^cd^	12.82 ^bcd^	12.35 ^bcd^	11.89 ^bcd^	11.06 ^cd^	10.90 ^d^	12.20 ^bcd^	0.81	0.01

Means within a row with different letters differ among different dietary treatments (*p* < 0.05), *n* = 6. DE, digestible energy; ME, metabolizable energy; DM, dry matter; EE, ether extract; NDF, neutral detergent fiber; ADF acid detergent fiber; CP, crude protein; GE, gross energy.

**Table 6:** Nutrient digestibility of cereal processing by-products for growing pigs.

Items	Wheat Middlings			Rice Polish		Rice Bran					Wheat Bran			Corn Germ Meal		SEM	*p*-Value
	1	2	3	1	2	1	2	3	4	5	1	2	3	1	2		
GE	80.67 ^b^	85.87 ^a^	85.86 ^a^	86.10 ^a^	87.71 ^a^	75.61 ^c^	82.91 ^ab^	74.00 ^c^	73.13 ^c^	61.57 ^e^	67.08 ^d^	64.84 ^de^	60.73 ^e^	60.77 ^e^	67.16 ^d^	1.24	0.01
DM	81.28 ^b^	90.24 ^a^	84.42 ^b^	90.68 ^a^	90.59 ^a^	72.44 ^c^	82.16 ^b^	66.13 ^de^	59.49 ^f^	56.33 ^f^	67.04 ^cd^	66.30 ^de^	58.14 ^f^	62.45 ^ef^	67.75 ^cd^	1.32	0.01
NDF	64.33 ^bc^	77.60 ^ab^	52.98 ^de^	80.31 ^a^	78.88 ^a^	50.85 ^de^	58.35 ^cd^	60.49 ^cd^	48.23 ^de^	57.13 ^cd^	48.09 ^de^	55.61 ^cd^	42.03 ^e^	56.06 ^cd^	71.33 ^ab^	3.24	0.01
ADF	43.88 ^bcd^	45.05 ^bc^	46.40 ^abc^	48.50 ^ab^	44.16 ^bcd^	34.88 ^de^	42.56 ^bcd^	34.86 ^de^	31.33 ^d^	57.41 ^a^	27.46 ^d^	30.04 ^d^	31.91 ^d^	51.59 ^ab^	55.81 ^a^	3.12	0.01
CP	84.71 ^bc^	94.48 ^a^	88.60 ^ab^	96.09 ^a^	95.42 ^a^	63.35 ^ef^	87.19 ^b^	88.16 ^ab^	44.69 ^f^	59.17 ^ef^	73.76 ^d^	80.48 ^bc^	72.20 ^d^	53.64 ^f^	78.56 ^cd^	2.06	0.01

Means within a row with different letters differ among different dietary treatments (*p* < 0.05), *n* = 6. DM, dry matter; NDF, neutral detergent fiber; ADF acid detergent fiber; CP, crude protein; GE, gross energy.

**Table 7:** Correlations between the chemical composition and available energy (as-fed basis) of cereal by-products for growing pigs.

Items	DM	Ash	EE	NDF	ADF	CP	GE	DE	ME
DM	1.00								
Ash	0.14	1.00							
EE	0.36	0.44	1.00						
NDF	0.50	−0.08	−0.26	1.00					
ADF	0.63 *	0.25	0.02	0.91 *	1.00				
CP	0.18	0.09	−0.18	0.71 *	0.64 *	1.00			
GE	0.66 *	0.29	0.91 *	0.07	0.29	0.05	1.00		
DE	−0.42	−0.05	0.29	−0.83 *	−0.86 *	−0.46	0.08	1.00	
ME	−0.41	−0.11	0.29	−0.82 *	−0.86 *	−0.48	0.10	0.99 *	1.00

* represents a significant difference (*p* < 0.05). DE, digestible energy; ME, metabolizable energy; DM, dry matter; EE, ether extract; NDF, neutral detergent fiber; ADF acid detergent fiber; CP, crude protein; GE, gross energy.

**Table 8:** Prediction equations for digestible and metabolizable energy in cereal by-products fed to growing pigs.

Items	Prediction Equations	R^2^	C (p)	AIC	BIC	*p*-Value
Developed by using energy contents and chemical composition (as-fed basis)						
1	DE (MJ/kg) = −0.4110 × ADF (%) + 16.1184	0.74	14.16	93.21	92.15	0.01
2	DE (MJ/kg) = −0.4597 × ADF (%) + 0.5988 × GE (MJ/kg) + 6.0138	0.86	4.86	90.25	91.02	0.01
3	ME (MJ/kg) = 1.0440 × DE (MJ/kg) − 1.1235	0.98	0.89	89.14	90.25	0.01
Developed by using energy contents and chemical composition (DM basis)						
1	DE (MJ/kg DM) = −0.0973 × NDF (%) + 16.7654	0.53	20.35	95.34	93.67	0.01
2	DE (MJ/kg DM) = −0.1451 × NDF (%) + 0.3025 × CP (%) +13.8595	0.72	15.86	91.34	92.02	0.01
3	ME (MJ/kg DM) = 1.0679 × DE (MJ/kg DM) − 1.46	0.98	2.35	90.79	91.43	0.01
4	ME (MJ/kg DM) = 1.1155 × DE (MJ/kg DM) + 0.0363 × ADF (%) − 2.3412	0.99	0.73	90.24	91.18	0.01

The prediction equations for DE and ME were developed on an as-fed basis. DE, digestible energy; ME, metabolizable energy; ADF acid detergent fiber; GE, gross energy; R^2^, coefficient of determination; C (p), Mallows’Cp; AIC, Akakike information criterion; and BIC, Bayesian information criterion.

## Data Availability

Data are contained within the article.
